# Fosfomycin susceptibility testing and resistance mechanisms in Enterobacterales in South Africa

**DOI:** 10.4102/ajlm.v13i1.2252

**Published:** 2024-03-19

**Authors:** Jessica S. Hurwitz, Mae Newton-Foot, Kristien Nel van Zyl, Pieter Nel

**Affiliations:** 1 Department of Pathology, Faculty of Medicine and Health Sciences, Stellenbosch University, Cape Town, South Africa; 2 Department of Medical Microbiology, Tygerberg Hospital, National Health Laboratory Service, Cape Town, South Africa

**Keywords:** fosfomycin, phenotypic susceptibility testing, molecular resistance mechanisms, *Escherichia coli*, *Klebsiella pneumoniae*, Enterobacterales

## Abstract

**Background:**

Fosfomycin treatment of urinary tract infections is increasingly attractive due to escalating antibiotic resistance rates among urinary pathogens. Standard antibiotic susceptibility testing methods perform poorly for fosfomycin as there is poor correlation between susceptibility results and clinical outcomes in urinary pathogens other than *Escherichia coli*.

**Objective:**

We evaluated the performance of fosfomycin susceptibility testing in *E. coli* and *Klebsiella pneumoniae* to determine whether fosfomycin susceptibility is associated with molecular resistance mechanisms.

**Methods:**

Forty-six each of *E. coli* and *K. pneumoniae* clinical isolates were obtained from a tertiary hospital in South Africa, from 01 June 2017 to 31 January 2018. Agar dilution, disk diffusion, and gradient diffusion were performed and interpreted using the Clinical Laboratory Standards Institute and European Committee on Antimicrobial Susceptibility Testing guidelines. Molecular resistance mechanisms were identified by whole genome sequence analysis.

**Results:**

Disk diffusion and gradient diffusion were accurate alternatives for fosfomycin susceptibility testing in *E. coli* (98% categorical agreement), but not in *K. pneumoniae* (47% categorical agreement). All *E. coli* isolates contained at least one resistance mechanism, but only one isolate with a *fos*A gene was resistant. In *K. pneumoniae,* 63% (29/46) and 70% (32/46) of isolates were susceptible to fosfomycin, using Clinical Laboratory Standards Institute and European Committee on Antimicrobial Susceptibility Testing breakpoints, respectively, despite all isolates containing a *fos*A gene and a *uhp*T mutation.

**Conclusion:**

A better understanding of fosfomycin susceptibility and improved antibiotic susceptibility testing tools could improve diagnostic capability and clinical guidelines for fosfomycin treatment of urinary tract infections.

**What this study adds:**

This study highlights the importance of adhering to interpretive guidelines when performing antimicrobial susceptibility testing and the need for simplified, accurate and standardised susceptibility testing methodology and interpretation for fosfomycin in Enterobacterales organisms.

## Introduction

Urinary tract infections (UTIs) caused by Gram-negative and Gram-positive bacteria, including those of the Enterobacterales order and *Enterococcus* species, pose a global burden, with more than 404 million individuals being affected in 2019.^1,2^ The most common causes of lower uncomplicated UTIs are *Escherichia coli* and *Klebsiella pneumoniae*,^3^ with *E. coli* causing 70% – 95% of upper and lower UTIs.^4,5^

Due to increased antibiotic resistance among urinary pathogens to more commonly used antibiotics and concerns with the side-effect profiles, alternative treatment options for UTIs are being assessed.^6,7^ Fosfomycin, an old antibiotic with broad-spectrum bactericidal activity against most urinary pathogens, is an excellent empiric choice for the treatment of lower uncomplicated UTIs.^8,9,10^

Fosfomycin acts by binding to uridine-diphosphate-N-acetylglucosamine enolpyruvyl transferase (MurA)^11^ to prevent the production of N-acetylmuramic acid, a peptidoglycan precursor.^12^ This inhibits peptidoglycan formation, leading to bacterial cell lysis and cell death.^13^ Fosfomycin resistance is mediated by modification of the target MurA protein, direct modification of fosfomycin by Fos enzymes,^14^ or by mutations in the glycerol-3-phosphate transport and the hexose phosphate uptake transport systems and their regulators, which are responsible for fosfomycin uptake.^8^

There is limited data in South Africa on fosfomycin susceptibility in *E. coli* and other Enterobacterales due to a lack of consensus in the interpretation of susceptibility results by the Clinical and Laboratory Standards Institute (CLSI) and the European Committee on Antimicrobial Susceptibility Testing (EUCAST), limited research, and a lack of information regarding associations between susceptibility test results, molecular mechanisms of resistance, and clinical outcomes.^9^ Nevertheless, *E. coli* fosfomycin resistance rates were reported to be as low as 3.2% in Europe, Asia and the United States between 2004 and 2009, with studies done after 2010 only suggesting a slight increase in resistance rates.^15^ In South Africa, two studies in 2020 and 2022 described fosfomycin resistance rates of 4.3% – 4.5% in UTI isolates and 2% – 8% in Enterobacterales.^3,16^

The CLSI and EUCAST guidelines provide fosfomycin susceptibility breakpoints for *E. coli* but the only breakpoints available for other Enterobacterales are agar dilution minimum inhibitory concentration (MIC) breakpoints from EUCAST, which are the same as those for *E. coli.* This is due to insufficient clinical and MIC data for establishing both clinical breakpoints and an epidemiological cut-off value. Due to the absence of breakpoints for other Enterobacterales, *E. coli* breakpoints have been used for interpretation of fosfomycin susceptibility.^3,15,16^ The inappropriate interpretation of fosfomycin susceptibility for *K. pneumoniae* may, however, lead to unfavourable clinical outcomes when the agent is used therapeutically.^8,17,18^

This study aimed to describe the association between phenotypic susceptibility to fosfomycin and molecular mechanisms of resistance to fosfomycin in both *E. coli* and *K. pneumoniae*.

## Methods

### Ethical considerations

Ethical approval was obtained from the Stellenbosch University Health Research Ethics Committee (N14/06/069, U22/05/180). A consent waiver was received since the isolates used were anonymised, the patients would neither be harmed nor benefit from the study directly, and no patient information was included in this study. The research data were stored electronically on a password-protected device accessible by the principal investigator only.

### Isolate collection

Forty-six each of *E. coli* and *K. pneumoniae* previously isolated from patient blood cultures (BacT/ALERT^®^ Culture Media, bioMérieux, Marcy-l’Étoile, France) at a tertiary hospital in South Africa between 01 June 2017 and 31 January 2018 as part of routine patient management were collected. Species identification was done as part of routine diagnostic procedures using the Vitek^®^2 automated system (bioMérieux, Marcy-l’Étoile, France).

### Fosfomycin susceptibility testing

Agar dilution was performed by spotting 1 μL of a 1:4 dilution of a 0.5 MacFarland suspension of each bacterial isolate on Mueller-Hinton agar plates (Sigma Aldrich, St. Louis, Missouri, United States) containing glucose-6-phosphate and fosfomycin (Fosfomycin sodium VETRANAL^®^, Sigma Aldrich, St. Louis, Missouri, United States) at a range of concentrations (1 μg/mL – 256 μg/mL), in two-fold increments.^17^ Agar dilution was performed in triplicate, and the MIC was determined as the lowest antibiotic concentration at which bacterial growth was not observed in at least two replicates following incubation at 37 °C for 16 h in ambient air.^19^

Gradient diffusion was performed using fosfomycin MIC strips (0.064 μg/mL – 1024 μg/mL) (Liofilchem, Roseto degli Abruzzi, Italy) on Mueller-Hinton agar plates (Green Point Media, Cape Town, South Africa) inoculated with a 0.5 McFarland bacterial suspension and incubated at 37 °C for 16 h in ambient air. The MIC was interpreted at the concentration where the zone of the ellipse intercepted with the strip.^20^

Kirby Bauer disk diffusion was performed using fosfomycin/glucose-6-phosphate discs (200 μg) (Mast Group, Bootle, United Kingdom) on Mueller-Hinton agar plates inoculated with a 0.5 McFarland bacterial suspension. The zone of inhibition (ZOI) was measured following incubation at 37 °C for 16 h in ambient air.

Quality control was performed using the fosfomycin susceptible *E. coli* American Type Culture Collection 25922 strain (ATCC, Manassas, Virginia, United States), with an MIC range of 0.5 μg/mL – 2 μg/mL and a ZOI range of 22 mm – 30 mm, as specified by CLSI.^17^

The antibiotic susceptibility testing results were analysed using the interpretive categories and breakpoints established by the CLSI (2020) and EUCAST (2020).^17,18^ For *E. coli,* CLSI interprets ZOI ≥ 16 mm/MIC ≤ 64 μg/mL as susceptible, ZOI = 13 mm/MIC – 15 mm/MIC = 125 μg/mL as intermediate, and ZOI ≤ 1 mm/MIC ≥ 256 μg/mL as resistant; due to the absence of breakpoints for *K. pneumoniae, E. coli* breakpoints were used for interpretation. For *E. coli,* EUCAST interprets ZOI ≥ 24 mm/MIC ≤ 32 μg/mL, as susceptible and ZOI < 24 mm/MIC > 32 μg/mL as resistant, but for *K. pneumoniae*, there are only agar dilution breakpoints; therefore *E. coli* disk diffusion breakpoints were used for interpretation. For disk diffusion, EUCAST recommends disregarding isolated colonies that are within the ZOI, while CLSI advises taking them into account.^17,18^ For gradient diffusion, the manufacturer’s guidelines suggest different approaches for *E. coli* and *K. pneumoniae*: for *E. coli*, single colonies in the zone of the ellipse should be disregarded, while for *K. pneumoniae*, colonies within 3 mm of the strip should be considered for both CLSI and EUCAST interpretations.^20^

### Fosfomycin resistance mechanisms

#### Whole genome sequencing analysis

Whole genome sequencing and genome assembly were previously performed on the *E. coli* and *K. pneumoniae* isolates using the Illumina MiSeq platform (Illumina, Inc., San Diego, California, United States).^21^

Resistance Gene Identifier version 5.2.1 (McMaster University, South Hamilton, Ontario, Canada) was used to identify fosfomycin resistance mechanisms in the assemblies, including *fos*A genes and the multidrug transporter *mdt*G, and mutations in the target gene *mur*A, the *uhp*T and *glp*T transporter genes, and the *cya*A and *pts*I regulator genes, using the Comprehensive Antibiotic Resistance Database (McMaster University, South Hamilton, Ontario, Canada).^22^ Outputs were generated for 95% identity.

The Artemis genome browser version 18.2.0 (Wellcome Sanger Institute, Hinxton, Cambridgeshire, United Kingdom)^23^ was used to manually view and curate the gene sequences to confirm the presence of the fosfomycin resistance mechanisms in a subset of assemblies.

### Data analysis

The performance of disk diffusion and gradient diffusion relative to the gold standard method, agar dilution, was described using categorical agreement, essential agreement (for gradient diffusion), and minor, major, and very major error rates. Categorical or essential agreement of greater than 90% and minor, major, and very major error rates of less than 3% were considered acceptable.^24^ The combinations of mutations and genes detected by whole genome sequencing analysis were compared to gold standard agar dilution MICs. Data were stored, and statistical calculations were done, in Microsoft^®^ Excel^®^ for Microsoft 365, 2022 (Microsoft^®^, Redmond, Washington, United States).

## Results

### Fosfomycin susceptibility

According to the gold standard method, agar dilution, 98% (45/46) of the *E. coli* isolates were susceptible to fosfomycin, according to both CLSI and EUCAST guidelines. Of the *K. pneumoniae* isolates, 70% (32/46) were susceptible, 22% (10/46) resistant, and 9% (4/46) intermediate, according to CLSI breakpoints, while 63% (29/46) of the *K. pneumoniae* isolates were susceptible and 37% (17/46) were resistant according to EUCAST breakpoints.

For *E. coli,* gradient diffusion showed 100% categorical agreement with agar dilution using EUCAST breakpoints, with one major error (2%) when interpreting the MICs according to CLSI breakpoints (Table 1). The essential agreement between gradient diffusion and agar dilution for *E. coli* was only 11%. Gradient diffusion did not perform well against agar dilution for *K. pneumoniae*, with a categorical agreement of around 75% depending on the guidelines used and an essential agreement of only 65%. Disk diffusion showed a categorical agreement of 100% against agar dilution using both CLSI and EUCAST breakpoints for *E. coli*. Disk diffusion did not perform well for *K. pneumoniae,* with a categorical agreement of 46% using EUCAST and 82% using CLSI breakpoints.

**TABLE 1:** Performance of fosfomycin gradient diffusion and disk diffusion susceptibility testing in comparison to agar dilution for 92 clinical isolates from South Africa (2017–2018) interpreted using the Clinical Laboratory Standards Institute (2020) and European Committee on Antimicrobial Susceptibility Testing (2020) breakpoints.

Susceptibility testing method	*Escherichia coli* (*n* = 46)		*Klebsiella pneumoniae* (*n* = 46)	
	CLSI	EUCAST	CLSI	EUCAST
**Gradient diffusion**				
Categorical agreement (%)	98	100	76	74
Minor error (%)	0	0	15	0
Major error (%)	0	0	7	20
Very major error (%)	2	0	2	7
Essential agreement (%)	11	-	65	-
**Disk diffusion**				
Categorical agreement (%)	100	100	82	46
Minor error (%)	0	0	13	0
Major error (%)	0	0	4	54
Very major error (%)	0	0	2	0

CLSI, Clinical Laboratory Standards Institute; EUCAST, European Committee on Antimicrobial Susceptibility Testing.

### Mechanisms of fosfomycin resistance

Various fosfomycin resistance mechanisms were detected in *E. coli*, with all the isolates carrying the *mdt*G gene and 93% (43/46) having the E448K mutation in the *glp*T gene (Table 2). Mutations in *uhp*T and the regulator genes, *cya*A and *pts*I, were also detected. A *fos*A gene was only detected in one *E. coli* isolate; this isolate had the highest fosfomycin MIC at greater than 256 μg/mL and was the only *E. coli* isolate classified as fosfomycin resistant. In the absence of *fosA*, MICs ranged from 1 μg/mL to 16 μg/mL regardless of the presence of different combinations of *glp*T, *uhp*T, and *cya*A mutations, and the presence or absence of *mdt*G genes (Table 3).

**TABLE 2:** Fosfomycin resistance mechanisms in 92 clinical isolates from South Africa (2017–2018).

Resistance mechanism	*Escherichia coli* (*n* = 46)		*Klebsiella. pneumoniae* (*n* = 46)	
	*n*	%	*n*	%
***fos*A (any)**	1	2	46	100
*fos*A3	1	2	0	0
*fos*A5	0	0	6	13
*fos*A6	0	0	40	87
*uhp*T (E350Q)	21	46	46	100
*glp*T (E448K)	43	93	0	0
*pts*I (V25I)	13	28	0	0
*cya*A (S352T)	16	35	0	0
*mdt*G	44	96	0	0
*mdt*G x2†	2	4	0	0

† , *mdt*G x2 indicates the presence of two *mdt*G genes.

**TABLE 3:** Combinations of fosfomycin resistance mechanisms and minimum inhibitory concentration distributions for 92 clinical isolates from South Africa (2017–2018).

Mechanisms of resistance	*n*	%	MIC range (μg/mL)
***Escherichia coli* (*n* = 46)**			
*fos*A3 + *glp*T + *cya*A + *mdt*G	1	2	> 256
*glp*T + *mdt*G	12	26	1–16
*uhp*T + *mdt*G	3	7	1–2
*glp*T + *cya*A + *mdt*G	11	24	1–8
*glp*T + *cya*A + *mdt*Gx2	1	2	4
*uhp*T + *glp*T + *cya*A + *mdt*G	3	7	1–8
*uhp*T + *glp*T + *pts*I + *mdt*G	13	28	1–8
***Klebsiella pneumoniae* (*n* = 46)**			
*fos*A6 + *uhp*T	40	87	1 to > 256
*fos*A5 + *uhp*T	6	13	8 to > 256

Note: *glp*T, *uhp*T, *cya*A and *pts*I represent the presence of mutations in these genes; *fos*A3, *fos*A5, *fos*A6 and *mdt*G represent the presence of a resistance gene; *mdt*G x2 indicates the presence of two *mdt*G genes.

MIC, minimum inhibitory concentration.

All of the *K. pneumoniae* isolates had a *fos*A gene, either *fos*A5 (6/46, 13%) or *fos*A6 (40/46, 87%), and the E350Q mutation in *uhp*T (Table 2). Both combinations were present in susceptible and resistant isolates with MICs that ranged from 1 μg/mL to 256 μg/mL (Table 3).

## Discussion

In this study, disk diffusion and gradient diffusion performed well in comparison to the gold standard agar dilution^25^ for *E. coli* using both CLSI and EUCAST guidelines. However, in the absence of interpretative criteria for other Enterobacterales species, neither method performed adequately for *K. pneumoniae*. Fosfomycin resistance mechanisms were identified in all *E. coli* and *K. pneumoniae* isolates, regardless of MIC, highlighting further challenges associated with fosfomycin susceptibility testing.

Regardless of the interpretive guideline used, all the tests in this study, except gradient diffusion, identified a single fosfomycin-resistant *E. coli* isolate. This low fosfomycin resistance rate (2%) is in keeping with the findings of previous studies.^3,15,16,26^ Gradient diffusion interpreted using CLSI breakpoints failed to identify the one resistant isolate, and the essential agreement between agar dilution and gradient diffusion for *E. coli* was low.

The manufacturers’ guidelines for interpreting single colonies within the zone of ellipse of gradient diffusion strips vary. Liofilchem recommends that for *E. coli*, single colonies should be ignored when measuring the zone of the ellipse, regardless of whether the CLSI or EUCAST guidelines are used for interpretation.^20^ Also, the CLSI and EUCAST breakpoints differ,^17,18^ influencing the interpretation. The different interpretations of MIC values did not greatly influence the susceptibility interpretation in *E. coli,* resulting in an acceptable categorical agreement of 98% according to CLSI (with a very major error rate of 2%) and 100% according to EUCAST guidelines. For *E. coli*, disk diffusion showed 100% categorical agreement with agar dilution using both CLSI and EUCAST guidelines, although EUCAST advises ignoring isolated colonies in the ZOI, and CLSI advises considering them.^17,18^

For *K. pneumoniae,* there were conflicting susceptibility results when interpreted using the two guidelines, CLSI and EUCAST, even for the gold standard agar dilution method. Although the variation in MIC values was lower when comparing gradient diffusion to agar dilution in *K. pneumoniae* than in *E. coli*, neither essential nor categorical agreement was acceptable. Similarly, the categorical agreement between agar dilution and disk diffusion, interpreted using both CLSI (82%) and EUCAST (46%) guidelines, was unacceptable. This is suspected to be due to the extrapolation of the guidelines provided by CLSI and EUCAST for *E. coli*, to *K. pneumoniae*.^17,18^ There were more single colonies within the inhibition zones in *K. pneumoniae* than *E. coli*, making it challenging to interpret the susceptibility results. Liofilchem advises that for *K. pneumoniae*, isolated colonies within the zone of the ellipse of the MIC strips should be considered irrespective of the interpretation guideline,^20^ and this contributed to the higher essential agreement for *K. pneumoniae* compared to *E. coli.* The lower categorical agreement for disk diffusion was interpreted using EUCAST guidelines, disregarding isolated colonies within the ZOI.

Based on these findings, neither gradient nor disk diffusion should be used as alternatives to agar dilution for fosfomycin susceptibility testing in *K. pneumoniae*. Furthermore, in agreement with this study, Bijllaardt et al. concluded that the breakpoints for *E. coli* should not be used for *K. pneumoniae*.^27^

Although various mutations associated with fosfomycin resistance in *E. coli* were detected in the *E. coli* isolates, no *mur*A mutations were detected, and only one isolate containing a *fos*A gene was fosfomycin resistant. Raised fosfomycin MICs were not detected in *E. coli* isolates with various combinations of fosfomycin resistance-associated mutations. A study by Doesschate et al. also found that *E. coli* isolates that did not have *fos*A genes were susceptible to fosfomycin.^28^

All of the *K. pneumoniae* isolates had a *fos*A gene (*fos*A5 or *fos*A6) and the E350Q mutation in the *uhp*T gene. The presence of these resistance mechanisms however had no correlation with the fosfomycin MICs of the the different isolates. It is, therefore, unclear whether these mechanisms contribute to fosfomycin resistance in this organism. The differential expression of *fos*A may account for the single colonies observed within the ZOI in *K. pneumoniae* isolates.^29^

Susceptibility tests for fosfomycin in *K. pneumoniae* are unreliable, with no clear association between phenotypic susceptibility and the presence of resistance mechanisms. The gold standard agar dilution test produced different results depending on the interpretive guidelines used, and there was low agreement between gradient diffusion or disk diffusion and agar dilution.

### Limitations

Although both gradient diffusion and disk diffusion, interpreted using CLSI and EUCAST guidelines, produced an acceptable categorical agreement for *E. coli*, a more extensive sample set of isolates containing more fosfomycin-resistant isolates, and with MICs around the breakpoints should be analysed to confirm these findings.

The aim of the study was to correlate the fosfomycin resistance mechanisms with the phenotypic susceptibility results. However, due to the very low prevalence of fosfomycin resistance amongst *E. coli* isolates, with only one resistant isolate, we were unable to test the significance of any association. Similarly, it was clear that there was no association between fosfomycin MIC and resistance mechanisms in *K. pneumoniae*.

Not all fosfomycin resistance genes or mutations may be included in the Comprehensive Antibiotic Resistance Database^22^, and therefore, mutations or genes that confer resistance could have been missed. Thus, whole genome sequence data should be manually searched to identify potential novel fosfomycin resistance mutations, which could be further validated to improve our understanding of fosfomycin susceptibility.

The expression of the resistance mechanisms, specifically the *fos*A genes, was not evaluated in this study. Differential expression may account for the variable MICs and susceptibility results despite the presence of these genes in all *K. pneumoniae* isolates.

### Conclusion

Fosfomycin molecular resistance mechanisms were not associated with phenotypic susceptibility to fosfomycin in *E. coli* or *K. pneumoniae*, except *fos*A3 in one resistant *E. coli* isolate. Disk diffusion and gradient diffusion perform well for fosfomycin susceptibility testing for *E. coli* but not for *K. pneumoniae*. These findings support the guidelines given by CLSI and EUCAST in that disk diffusion and gradient diffusion susceptibility testing should not be performed for *K. pneumoniae* and that the *E. coli* breakpoints should not be used for interpreting *K. pneumoniae* susceptibility.
